# Development of a Shortened Version of the Nurse Managers’ Empowering Behavioral Scale for Staff Nurses

**DOI:** 10.3390/healthcare10102112

**Published:** 2022-10-21

**Authors:** Miki Sasaki, Yasuko Ogata, Keiko Fujinami, Yuki Yonekura

**Affiliations:** 1 Department of Gerontological Nursing and Healthcare Systems Management, Graduate School of Health Care Sciences, Tokyo Medical and Dental University, Tokyo 113-8510, Japan; 2 Department of Nursing Informatics, Graduate School of Nursing Science, St. Luke’s International University, Tokyo 104-0044, Japan

**Keywords:** empowerment, leadership, measurement, nurse, reliability, validity

## Abstract

The original version of the Nurse Managers’ Empowering Behavioral Scale for Staff Nurses (NMEB-SN) was both unique and comprehensive. However, it was considered lengthy. Hence, the intention of this particular study was to develop a shortened version of the NMEB-SN comprising 15 items across five subscales. Responses from 1268 staff nurses working at 10 Japanese hospitals were included in the analysis to confirm the validity and reliability of the scale. The results ensured internal consistency, construct and criterion-related validity, and test–retest reliability. The NMEB-SN short version is applicable in the context of practical and efficient nursing management to attract more nurses to the workplace.

## 1. Introduction

The concept of psychological empowerment was developed as a component of motivation [1,2] and defined by Spreitzer [3] as a “motivational construct” consisting of meaning, competence, self-determination, and impact. It is important to psychologically empower staff nurses so that they can continue to work and fulfill their roles. The factors associated with the psychological empowerment of employees include individual characteristics such as positive self-evaluation traits, as well as contextual factors such as leadership and social-political support [4]. Empowering leadership, a type of leadership style, psychologically empowers employees and is associated with increased work engagement and organizational commitment, and a reduced intention to leave [5,6,7,8,9].

The Nurse Managers’ Empowering Behavioral Scale for Staff Nurses (NMEB-SN) [10] was developed based on interviews with staff nurses regarding psychologically empowering and motivating behaviors toward staff nurses. The NMEB-SN comprises 48 items across five subscales—providing meaning to work; supporting autonomy to make me have self-confidence; providing support to overcome obstacles at work; recognizing work; and respecting me as a staff member. This scale is unique in that it is specific to nurse managers and includes the component “recognizing work,” which is not found in previously developed empowering leadership scales [11,12,13,14,15]. If nurse managers can demonstrate leadership by utilizing scales with these components, it may help revitalize the nursing field. However, the NMEB-SN has 48 items, making it not only effort-intensive but also time-consuming to complete. This can lead to poor response rates and poor-quality answers [16,17]. A scale with fewer items may contribute to the advancement of research on nurse managers’ leadership. Therefore, this study created the short version of the NMEB-SN and confirmed its reliability and validity.

## 2. Materials and Methods

### 2.1. Process of Scale Item Reduction

The minimum number of items that would ensure reliability with content validity of each subscale was set at three. To identify the items to be included in the NMEB-SN short version, three nursing management researchers worked together in this study to select three items from each subscale (Table 1). For each subscale, (a) items with the highest correlation with the remaining total score, (b) items with the highest face validity, and (c) items that most represent the construct [18] were selected.

### 2.2. Data Sources and Participants

This study utilized the data [10] that was used to develop the NMEB-SN. Questionnaires were administered to 2325 staff nurses in 10 Japanese hospitals from December 2018 to January 2019, with 1516 responses collected. The data of participants with missing items in the NMEB-SN, an unclear registered nurse license, and missing age and years of nursing experience related information were deleted. Finally, the data of 1268 staff nurses working in general wards were analyzed. Furthermore, of the data collected two–three weeks later from three out of ten hospitals, 219 responses were included in the analysis to confirm test–retest reliability. Participants in this phase of data collection responded to the initial survey, and there was no change in their nurse managers.

### 2.3. Instruments

Participants provided information regarding their demographic characteristics, the NMEB-SN items, and external criteria that were used in the criterion-related validity of the NMEB-SN. The NMEB-SN items were scored on a 5-point Likert scale, and the other external criteria items were scored on a 7-point Likert scale. A higher score indicated that nurse managers exhibit psychologically empowering and motivating behaviors toward staff nurses.

Leader–member exchange was evaluated using the 12-item multidimensional measure of leader–member exchange Japanese version (LMX-MDM-J) [19]. Higher scores indicate a higher quality relationship between the nurse manager and staff nurse.

Psychological empowerment was evaluated using the Japanese translation [20] of Spreitzer’s [3] 12-item psychological empowerment instrument, wherein higher scores suggest higher psychological empowerment of staff nurses.

Affective commitment was evaluated using the 3-item Affective Organizational Commitment Scale [21]. Higher scores indicate a stronger affective commitment of staff nurses to their organization.

Work engagement was evaluated using the 9-item Japanese version of the Utrecht Work Engagement Scale [22], wherein higher scores indicate higher work engagement of staff nurses.

Job satisfaction and turnover intention were individual items evaluated on the 7-point Likert scale, wherein higher scores denote higher job satisfaction and turnover intention.

### 2.4. Data Analysis

Following the NMEB-SN development procedure, the reliability and validity of the short version were verified. Internal reliability was verified by Cronbach’s alpha coefficient. To verify the test–retest reliability, an intra-class correlation coefficient (ICC) of the NMEB-SN short version was performed. Construct validity was verified by confirmatory factor analysis (CFA). Whether the model fit was acceptable was assessed by the comparative fit index (CFI) and root mean square error of approximation (RMSEA). Criterion-related validity was tested using Pearson’s correlations.

## 3. Results

**Figure 1:**
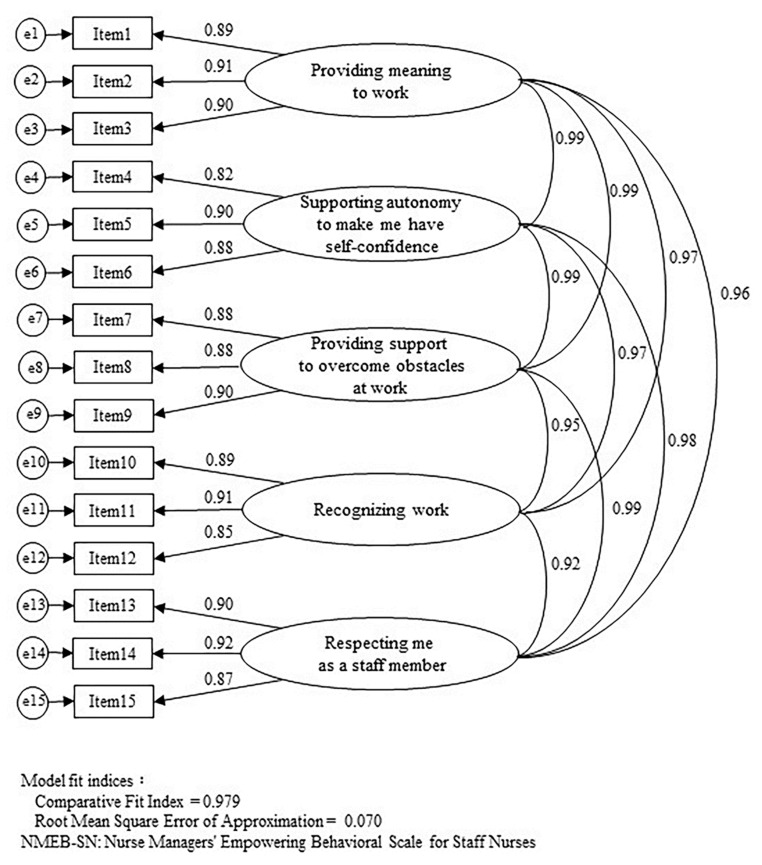
Confirmatory factor analysis of the NMEB-SN short version.

A total of 1268 participants were included in the study, with a mean age of 31.8 years (standard deviation: SD 8.97) and a mean years of experience of 8.52 (SD 7.95). Of the participants, 1151 (90.8%) were women, 1145 (90.3%) were staff who were not in the position of deputy nurse manager, and 915 (72.2%) were from a vocational school. Construct validity was assessed by the goodness-of-fit indices of CFA. The CFI value was 0.979 and the RMSEA value was 0.070 (90% confidence interval, CI: 0.065–0.076), which met the criteria for acceptable model fit (Figure 1). The Cronbach’s alpha coefficient of internal reliability was greater than 0.90, and the ICC value used for verifying the test–retest reliability was greater than 0.88 (Table 1). The Pearson’s correlation coefficients between the NMEB-SN short version and external criteria for assessing criterion-related validity were as high as 0.86 for the LMX-MDM, which captures nurse manager leadership in the quality of the staff nurse–nurse manager relationship; correlations with other variables ranged from moderate to weak (*p* < 0.001; Table 2).

## 4. Discussion

### 4.1. Validation of the NMEB-SN Short Version

As a result of implementing CFA, the model fit met the following criteria: CFI ≥ 0.90 and RMSEA < 0.08 [23]. Therefore, construct validity was confirmed. Cronbach’s alpha and the ICC between the two surveys exceeded the standard value of 0.70 [24], confirming internal reliability and test–retest reliability. The coefficients of the correlation analysis were almost the same in the results from the development of the NMEB-SN [10] and in the meta-analysis by Kim et al. [6], confirming its criterion-related validity. Thus, the efficacy of the NMEB-SN short version was verified.

### 4.2. Implications for Future Research

The shortened version of the scale was created using data from the development of the original version of the NMEB-SN. The NMEB-SN items were developed from the results of interviews conducted with staff nurses working in actual nursing workplaces. These items are characterized by the particular actions of the nurse managers that staff nurses recognize. As the shortened version is used in additional research, its reliability and validity will be characterized further.

The NMEB-SN shortened version can be used to examine and understand the relationship between the nurse managers’ empowerment behavior toward staff nurses and other variables, whereas the original long version can be used to investigate nurse managers’ behavior in detail. This should lead to nursing management that will attract more nurses to the workplace. The use of the scale might also encourage inter-organizational and international comparative studies.

## 5. Conclusions

The validity and reliability of the NMEB-SN short version, comprising 15 items across five subscales, were verified. The scale can be used to measure the empowerment provided by nurse managers’ behavior, which in turn can help psychologically empower and motivate staff nurses, resulting in improved performance and engagement in the workplace.

## Figures and Tables

**Table 1:** Items, descriptive statistics, Cronbach’s α (*n* = 1268), and ICC of the NMEB-SN short version (*n* = 219).

Item		Mean	SD	Cronbach’s α	ICC
Providing meaning to work		3.51	1.00	0.93	0.88
1.	My nurse manager explains things clearly and in a manner suited to my experience in order that I will understand				
2.	My nurse manager asks me to perform work in a way that makes me act positively				
3.	My nurse manager creates opportunities for me to think about what sort of nursing I should aim to achieve				
Supporting autonomy to make me have self-confidence		3.53	0.96	0.90	0.90
4.	My nurse manager reflects our suggestions in work				
5.	My nurse manager works to gain the understanding of those around me so I can perform my role at work				
6.	My nurse manager leaves self-determination in my work up to me, but takes final responsibility				
Providing support to overcome obstacles at work		3.42	1.01	0.91	0.90
7.	My nurse manager shows me new perspectives so that my work will go well				
8.	My nurse manager notices when I come across trouble at work, judges the right time and provides opportunities to discuss matters				
9.	My nurse manager supports my efforts to reflect upon issues in my approach my work				
Recognizing work		3.41	0.98	0.91	0.88
10.	My nurse manager tells me that I have matured regarding the way I perform my daily work				
11.	My nurse manager praises the results of my work				
12.	My nurse manager utilizes the results of the work I have done				
Respecting me as a staff member		3.57	1.03	0.92	0.89
13.	My nurse manager realizes when we are in an extremely difficult situation and shows empathy				
14.	My nurse manager tells me that s/he understands my position				
15.	My nurse manager listens to our opinions and thoughts about work				
Composite score		3.49	0.95	0.98	0.92

ICC: intra-class correlation coefficient, NMEB-SN: Nurse Managers’ Empowering Behavioral Scale for Staff Nurses, SD: standard deviation. Items were cited from the study by Sasaki, Ogata, Morioka, Yumoto, and Yonekura [10]. These items were first developed in Japanese and later translated into English.

**Table 2:** Descriptive statistics, Cronbach’s α, and Pearson’s correlation analysis of the NMEB-SN short version.

	*n*	Score Range	Mean	SD	Cronbach’s α	NMEB-SN Short Version					
						Composite Score	Providing Meaning to Work	Supporting Autonomy to Make Me Have Self-Confidence	Providing Support to Overcome Obstacles at Work	Recognizing Work	Respecting Me as a Staff Member
Leader–member exchange	1242	1–7	4.64	1.39	0.97	0.86	0.82	0.83	0.82	0.79	0.83
Psychological empowerment	1229	1–7	3.81	0.89	0.93	0.25	0.23	0.26	0.22	0.28	0.22
Affective commitment	1256	3–15	7.87	2.70	0.77	0.34	0.32	0.34	0.32	0.33	0.31
Work engagement	1232	0–54	20.02	9.49	0.94	0.26	0.25	0.25	0.24	0.26	0.24
Job satisfaction	1260	1–7	3.90	1.59	―	0.50	0.48	0.49	0.47	0.48	0.47
Turnover intention	1238	1–7	4.63	1.69	―	−0.36	−0.34	−0.35	−0.34	−0.34	−0.34

NMEB-SN: Nurse Managers’ Empowering Behavioral Scale for Staff Nurses, SD: standard deviation. For all correlations, *p* < 0.001. Pair-wise deletion.

## Data Availability

The datasets used and/or analyzed during the current study are available from the corresponding author on reasonable request.
